# Supplementary figures and images for: Lifestyle practices and associated factors among adults with hypertension: Conquering Hypertension in Vietnam-solutions at the grassroots level study
Source: PLoS One. 2024 Jun 6;19(6):e0303354. doi: 10.1371/journal.pone.0303354 (PMC11156363; doi:10.1371/journal.pone.0303354)

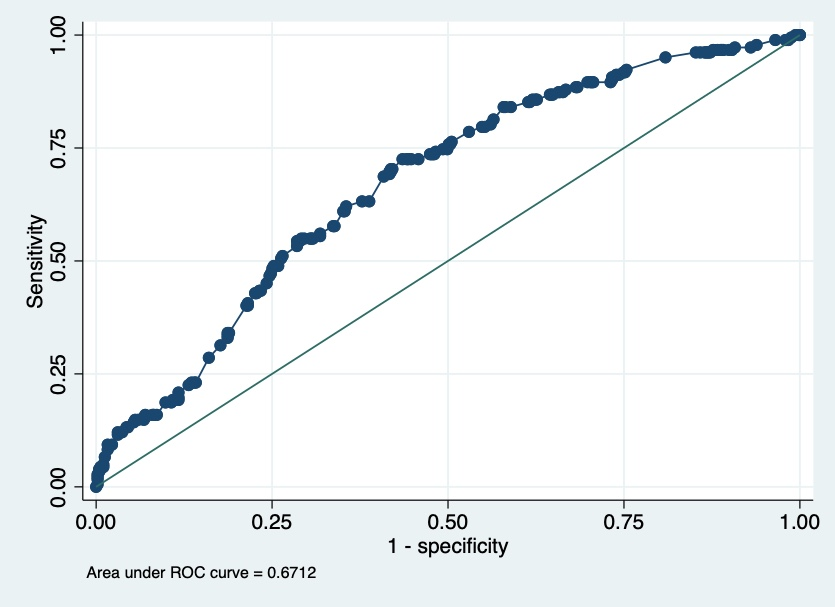

Supplement: S1 Fig — (TIF) [file pone.0303354.s001.tif]
