# Supplementary material for: Lifestyle practices and associated factors among adults with hypertension: Conquering Hypertension in Vietnam-solutions at the grassroots level study
Source: PLoS One. 2024 Jun 6;19(6):e0303354. doi: 10.1371/journal.pone.0303354 (PMC11156363; doi:10.1371/journal.pone.0303354)
Supplement: Attachment — Submitted filename: Response to Reviewer.docx [file pone.0303354.s002.docx]

**PONE-D-23-35910**

Lifestyle practices and associated factors among adults with hypertension in Vietnam: Conquering Hypertension in Vietnam-solutions at the grassroots level study

We thank both the Editors and reviewers for their comments. Below, we have attempted to address each of the reviewers’ comments in a point-by-point manner.

**Editor comments:**

1. *Stating the role of the funders.*

***Response:*** *The funders had no role in our study design, data collection and analysis activities, decision to publish, or preparation of the manuscript. We have addressed this issue in the cover letter.*

2. *Funding information: Remove funding-related text from the manuscript. Update funding statement.*

***Response:*** *We have removed this from the body of the manuscript and have updated the funding statement in our cover letter.*

## 3. *Data availability statement. Confirm at this time whether or not submission contains all raw data required to replicate the results of your study.*

*“De-identified participant data that were collected for this study can be obtained by contacting the corresponding author. Data, with an accompanying dictionary, will be made available, beginning with publication, after approval of a short proposal summarizing the analyses to be done.”*

*We have added this sentence at the end of the revised manuscript. (Line 338)*

**Reviewer #1:**

**SUMMARY:** This is a cross-sectional study that examines the prevalence of unhealthy lifestyle practices and associated factors among Vietnamese adults with uncontrolled hypertension. The data are extracted from the baseline of a cluster randomized trial study and the principal outcome is a binary index, obtained by dichotomizing the sum of unhealthy practices and regressed over a battery of covariates. Due to series of shortcomings, I’m afraid that the statistical analysis must be re-done (see the major issue below). I’m also appending a couple of specific issues about missing values and model selection that should be addressed by the authors.

**MAJOR ISSUE**

1. The principal outcome, unhealthy lifestyle practices, is summarized by a binary indicator (≤1 and ≥2 unhealthy practices). This approach is generally not recommended for several reasons. **First**, it assumes exchangeability between unhealthy practices: being a smoker and a heavy drinker is considered the same as begin physically inactive and with an unhealthy diet. **Second**, in the case of incomplete profiles (missing values; see specific issue no 1), there are not obvious ways to compute indicator. **Third**, it is based on the arbitrary threshold 2 (why not 1 or 3?). A simple way to overcome these difficulties is a logistic regression model where the four lifestyle practices are conditionally independent given subject-specific random effects and regression coefficients are outcome-specific. This kind of mixed effects model is available in all major statistical software platforms and would better support the discussion in the section “Prevalence of unhealthy lifestyle practices”.

***Response:*** *We thank the reviewer for their very helpful comment. Although the unhealthy lifestyle practices are not exchangeable, the total number of unhealthy practices remains important to understand. While there is an extensive literature examining factors associated with each unhealthy practice described in our paper, there is a lack of studies examining the number of unhealthy lifestyle practices. We chose 2 as the cut-off point for this study based on the distribution of this variable (****Table 1****)*

| **Number of unhealthy lifestyle practices** | **n** | **%** |
| --- | --- | --- |
| None | **120** | **17.9** |
| One | 368 | 54.8 |
| Two | 161 | 24.0 |
| Three | 20 | 3.0 |
| Four | 2 | 0.3 |

*We have also performed a multinominal logistic regression model for three category outcomes (0, 1, and >= 2* unhealthy lifestyle *practices) and the results were consistent with the results from the binominal logistic regression for the binary outcome (<=1 vs >=2) (****Tables below****). However, since the number of study participants with no unhealthy lifestyle practices was relatively small (n=120) in the present study, and frequency for some categories (eg., Educational level-* *College/University, Hypertension level III) were low, some* relative risk ratio*s (RRRs) yielded from the multinominal logistic regression model were not stable (with wide range). We, therefore, selected the binominal logistic regression model as the main analytical approach for this study. We have added the sentence below to the revised methods section.*

*“We also performed a multinominal regression model for the three-category outcome (having 0, 1, and* ≥ *2 unhealthy lifestyle practices). The results from this modelling approach were consistent with those from the binominal logistic regression for the binary outcome (* ≤ *1 vs* ≥*2). However, due to the relatively small sample size in the “0 unhealthy” group and low frequencies in some response categories, we combined participants with 0 and 1 unhealthy lifestyle. We reported results from the binominal logistic model.”. (Line 178)*

*We will, however, consider including these Tables as appendices if the Editors think that they will be useful for journal readers.*

**Table 2: Study participant’s characteristics according to the number of unhealthy lifestyle practices**

|  | **Number of unhealthy**  **lifestyle practices** | | | ***p*-value** |
| --- | --- | --- | --- | --- |
|  | **0** | **1** | **≥2** |  |
| Age (mean±SD) (years) | 66.2±8.9 | 66.3±8.7 | 68.1±9.5 | **0.061** |
| Age group (years) (n, %) |  |  |  |  |
| 25-54 | 11 (9.2) | 30 (8.1) | 9 (4.9) | **0.160** |
| 55-64 | 42 (35.0) | 115 (31.3) | 58 (31.7) |  |
| 65-74 | 43 (35.8) | 162 (44.0) | 70 (38.3) |  |
| ≥75 | 24 (20.0) | 61 (16.6) | 46 (25.1) |  |
| Male (n, %) | 40 (33.3) | 162 (44.0) | 100 (54.6) | **0.001** |
| Marital status (n, %) |  |  |  |  |
| With a partner | 93 (77.5) | 294 (79.9) | 143 (78.1) | 0.81 |
| Education level (n, %) |  |  |  |  |
| Primary school or less | 24 (20.0) | 90 (24.5) | 58 (31.9) | **0.110** |
| Secondary school | 66 (55.0) | 201 (54.8) | 87 (47.8) |  |
| High school | 18 (15.0) | 46 (12.5) | 29 (15.9) |  |
| College/University | 12 (10.0) | 30 (8.2) | 8 (4.4) |  |
| Occupation (n, %) |  |  |  |  |
| Employed | 59 (49.2) | 183 (49.7) | 65 (35.5) | **0.002** |
| Unpaid work/Unemployed | 36 (30.0) | 104 (28.3) | 83 (45.4) |  |
| Retired | 25 (20.8) | 81 (22.0) | 35 (19.1) |  |
| BMI category (n, %) |  |  |  |  |
| Underweight | 5 (4.2) | 18 (4.9) | 8 (4.4) | 0.59 |
| Normal | 48 (40.0) | 173 (47.0) | 95 (51.9) |  |
| Overweight | 38 (31.7) | 99 (26.9) | 43 (23.5) |  |
| Obese | 29 (24.2) | 78 (21.2) | 37 (20.2) |  |
| HTN level (n, %) |  |  |  |  |
| Level I | 56 (46.7) | 177 (48.1) | 76 (41.5) | **0.012** |
| Level II | 45 (37.5) | 156 (42.4) | 70 (38.3) |  |
| Level III | 19 (15.8) | 35 (9.5) | 37 (20.2) |  |

**Table 3: Factors associated with the number of unhealthy lifestyle practices: Results from a multinomial logistic regression model.**

|  | **RRR (95% CI) *** | |
| --- | --- | --- |
|  | **1** | **≥2** |
| Age group (years) (n, %) |  |  |
| 25-54 | 1.0 | 1.0 |
| 55-64 | 1.09 (0.49-2.41) | 1.63 (0.60-4.44) |
| 65-74 | 1.35 (0.59-3.09) | 1.32 (0.47-3.71) |
| ≥75 | 0.81 (0.31-2.14) | 1.05 (0.33-3.37) |
| Sex |  |  |
| Female | 1.0 | 1.0 |
| Male | **1.93 (1.20-3.09)** | **3.72 (2.17-6.39)** |
| Education level |  |  |
| Primary school or less | 1.0 | 1.0 |
| Secondary school | 0.56 (0.30-1.05) | **0.36 (0.18-0.72)** |
| High school | 0.46 (0.20-1.02) | **0.41 (0.17-0.99)** |
| College/University | 0.43 (0.17-1.09) | **0.18 (0.06-0.57)** |
| Occupation |  |  |
| Employed | 1.0 | 1.0 |
| Unpaid work/  Unemployed | 0.90 (0.52-1.54) | **2.11 (1.15-3.87)** |
| Retired | 0.99 (0.53-1.86) | 1.21 (0.58-2.52) |
| Hypertension level |  |  |
| Level I | 1.0 | 1.0 |
| Level II | 1.11 (0.70-1.74) | 1.15 (0.68-1.95) |
| Level III | **0.50 (0.26-097**) | 1.13 (0.57-2.23) |

The reference category in the multinomial logistic regression model is 0 unhealthy lifestyle practices.

*RRR = relative risk ratio

**SPECIFIC ISSUES**

1. Missing values. Nothing is said about the treatment of incomplete profiles. How were missing values treated?

***Response:*** *There were only 2 study participants who had missing values on the education variable because they refused to answer the question about their level of education achieved; these individuals were not included in the multivariable regression model. We added this information in the footnote to Table 2 (line 227).*

2. Model selection. The selection of the variables to be included in the model rely on the p-value threshold 0.2 in a univariate analysis (as said in line 170). This is not a recommended approach, because the p-value of a variable changes when further variables are included in the model, due to the correlation between covariates. More robust approaches rely on stepwise selection methods that rely on comparing the AIC values obtained before and after discarding a variable.

***Response:*** *The focus of the paper was on examining factors associated with the outcome and we used univariate analysis to screen variables for possible inclusion in our multivariable-adjusted regression analyses. We chose a p value of 0.2 as the cut-off point for the univariate analysis to avoid excluding factors that may have been potentially associated with the principal study outcome of the number of unhealthy lifestyle practices. Stepwise selection methods are very useful when independent variables are highly correlated. Independent variables in our study are not highly correlated.*

**Reviewer #2:**

Manuscript entitled “Lifestyle practices and associated factors among adults with hypertension in Vietnam: Conquering Hypertension in Vietnam-solutions at the grassroots level study” done by Phuong H. Nguyen et al. is interesting in the field of hypertension related to lifestyle. However, there are some major issues which should be clarified by the authors.

1. The number of included study subjects is small and there is only one province was selected for studying. Thus, the results could not be considered as representative for whole country. The authors should change the title and the conclusion statement.

***Response:*** *The “Conquering Hypertension in Vietnam-solutions at the grassroots level study” is the title of the parent study. We only mentioned this in our paper title to link our paper to the parent study. As requested, however, we have revised the conclusion statements in both the title, the abstract and the main body of the manuscript to reflect the reviewer’s concern. (Line 1, 50, 54, 332, and 336)*

2. Other included study subjects’ characteristics should be done in Table 1: Socio-economic status, Co-morbidities, Daily salt intake, Stress, and Sleep disturbances.

***Response:*** *Various sociodemographic characteristics and comorbidities are presented in Table 2 according to the number of unhealthy lifestyle practices. Data on stress or obstructive sleep apnea were not collected in the parent trial.*

3. Other causes of hypertension such as stress or obstructive sleep apnea should be excluded and discussed.

***Response:*** *While we agree with the reviewer’s suggestion, data on stress or obstructive sleep apnea were not collected in the parent trial.*

4. The P value for each OR should be done.

***Response:*** *We have added p-values in the revised manuscript (Table 3). (Line 240)*

5. The Figures of OR and ROC curve should be done to well illustrate the results.

***Response:*** *We have added the ROC figure in the revised manuscript (Figure 1). (Line 244)*

6. Sleep hygiene should be discussed as potential risk of hypertension and lifestyle modifiable factors.

***Response:*** *While we agree with the reviewer’s suggestion, data on sleep hygiene were not collected in the parent trial.*

**Minor issues**

1. Line 147-149: Socio-demographic, medical history, and clinical factors that were potentially associated with the primary study outcome included age group (25-54, 55-64, 67-74, and ≥75 years): lack of 65-66 years, please correct it.

***Response:*** *We have corrected this error in the revised manuscript. (Line 149)*

2. Data should be presented as mean ± SD or SE.

***Response:*** *Several continuous variables, such as the amount of time spent on physical activity during a typical week and number of fruit and vegetable servings consumed in an average day, (Table 1) were summarized using medians and the IQR since their distributions were skewed.*
